# Brain areas impaired in oral and verbal apraxic patients

**Published:** 2014-04-03

**Authors:** Fariba Yadegari, Mojtaba Azimian, Mahdi Rahgozar, Babak Shekarchi

**Affiliations:** 1 Department of Speech Therapy, University of Social Welfare and Rehabilitation Sciences, Tehran, Iran; 2 Department of Clinical Sciences, University of Social Welfare and Rehabilitation Sciences, Tehran, Iran; 3 Department of Statistics, University of Social Welfare and Rehabilitation Sciences, Tehran, Iran; 4 Department of Radiology, School of Medicine, AJA University, Tehran, Iran

**Keywords:** Broca’s Area, Insula, Left Hemisphere Damage, Oral Apraxia, Verbal Apraxia

## Abstract

**Background:** As both oral and verbal apraxia are related to vocal orofacial musculature, this study aimed at identifying brain regions impaired in cases with oral and verbal apraxia.

**Methods:** In this non-experimental study, 46 left brain damaged subjects (17 females) aged 23–84 years, were examined by oral and verbal apraxia tasks. Impaired and spared Broca’s area, insula, and middle frontal gyrus in the left hemisphere were checked from magnetic resonance imaging and computed tomography scans utilizing Talairach Atlas. Data were analyzed using chi-square test.

**Results:** Insula was significantly impaired in both forms of oral and verbal apraxia and different severities and prominent forms of both apraxias (P < 0.05). Broca’s area was slightly less involved than insula in two forms of apraxia.

**Conclusion:** As the damage of insula was more prominent in both forms of apraxias, it seems that oral and verbal apraxia may have commonalities regarding their underlying brain lesions.

## Introduction

The term “apraxia” is typically defined as an inability to plan and execute purposeful movements. Among different types of apraxia, there are two apparently related apraxias: oral and verbal apraxia, which occurs in relation to buccofacial musculatory motor planning. Oral apraxia is considered a higher order disorder of orofacial movements for non-speech gestures. It has been associated with inferior frontal, deep frontal white matter, insula, the posterior pars opercularis of the inferior frontal gyrus, the Rolandic operculum and basal ganglia lesions.^1^^-^^5^

Verbal apraxia also called apraxia of speech (AOS) is defined by Duffy^6^ as, “…a neurologic speech disorder that reflects an impaired capacity to plan or program sensorimotor commands necessary for directing movements that result in phonetically and prosodically normal speech.” (p. 307). Some investigations have been focused on brain areas involved in verbal apraxia. The most famous of them is that of Dronkers^7^ who concluded that anterior insula was the main impaired cite in verbal apraxia.

Verbal apraxia has also been reported to result following damage to Broca’s area,^8^ basal ganglia,^9^ insular and temporal regions, even right inferior frontal regions.^10^^,^^11^

As oral and verbal apraxia both are occurred in motor programming of the same orofacial apparatus, the question remains whether there are commonalities between brain’s areas involved in them. Hillis et al.^8^ questioned lesion overlap studies validity, discussing that the area of greatest overlap among large strokes may just reflect the vulnerability of the regions to ischemia, a point that was later addressed by Trupe et al.^12^ who raised the possibility that the association mentioned between anterior insula infarction and verbal apraxia might be accounted for by the fact that verbal apraxia generally persists in cases of large middle cerebral artery (MCA) strokes which always involve the insula. So to better address this issue, we decided to examine a control area (middle frontal gyrus [MFG]) which is also fed by MCA branches.

Our specific questions were: (1) Are Broca’s area, left insula and MFG impaired in patients with oral apraxia? (2) Are these areas impaired in patients with verbal apraxia? (3) Are involvement of these areas in various severities of apraxias different? (4) Which areas are more involved in subgroups of co-occurred apraxias? And (5) are there commonalities between areas involved in oral and verbal apraxia?

## Materials and Methods

***Participants***

Among 83 early examined patients, 55 patients met the inclusion and exclusion criteria and just 46 patients had magnetic resonance imaging (MRI) or computed tomography (CT) scans. These 46 brain damaged patients of which 17 females participated in this study. They were recruited from hospitals, private clinicians, and neuro-rehabilitation centers of Tehran, Karaj, Shiraz, and Mashad. All patients were native Farsi speakers with normal or corrected-to-normal hearing and vision. Participants were right handed and scored at the ceiling in the Edinburgh handedness inventory with unilateral left hemisphere lesions, and aged 23–84 (mean = 54 years). They were all literate with 1–119 month range of post onset time. Exclusion criteria included the presence of other neurological disease, severe auditory comprehension deficits, dementia or cognitive impairment, right hemisphere damage, and more than one cerebral accident.

This study was approved by the Research Ethics Committee of the University of Social Welfare and Rehabilitation Sciences, Tehran, Iran. All participants signed a free and informed consent form.

***Procedure***

*Apraxia tasks*

*All task sessions were videotaped by a Sony Digital Handy Cam (200M) for further analysis.

Diagnosis of oral apraxia

Oral apraxia was assessed by a task in which patients are asked to reproduce a variety of buccofacial gestures to verbal commands. This task has 22 items including instructions for tongue, lips, mouth, and vocal cords movements. The scoring procedure was as follows: 0 for a correct response, 1 for an erroneous response according to 14 predicted errors and 2 for no response. Total score was 44 representing the maximum error and the highest amount of apraxia (cut-off: 3). Lawsche’s content validity ratio of this task was above 50% for all items according to 25 experts opinions.^13^ Cut-off score was determined according to the fifth percentile of 102 healthy adults’ performance (aged 20–80 years). Cut-off score also was validated by clinical judgment as is described in severity ratings section.

Diagnosis of verbal apraxia

The verbal apraxia task was originally translated into Farsi and adapted from expressive speech subtest of Luria-Nebraska Neuropsychological Battery (LNNB)(I) to investigate adults brain neuropsychological profiles (Kapurkhani, 2007, unpublished MSc thesis). As it comprised oral expression parts and involved a scoring system similar to our oral apraxia task which rendered two apraxias comparable, it was adapted for verbal apraxia and used by the authors in this research. The task comprises 25 items in seven parts: repetition of speech sounds and words, telling the sound of letters, reading words, sentence repetition, reciting automatic series, story retelling and narrative speech. Scoring procedure was according to the number of errors in each section. We elucidate this scoring by the example of subtest of repetition of speech sounds. This subtest is administered as follows: “please repeat theses sounds: /e/, /â/, /m/, /d/, /ŝ/.” If the subject says all the sounds correctly the score will be (0). If (s)he has 1–2 errors, the score will be 1, and if 3–5 errors are produced, the score will be 2.

For the subtest of story retelling and narrative speech, the scoring comprised two criteria: time taken to start to respond and the number of words in the first 5 s. In story retelling, a story was read to the patients, while the passage was in front of him(her). Then (s)he was asked to retell the story. The time taken to start to respond was calculated. If this time was 0–10 s, the score was 0. If it was 11–22 s, the score was 1, and if it was 23–31 s, the score was 2. Also if the number of spoken words in the first 5 s was >9, the score was 0, if it was 6–9, the score was 1, and if it was 0–5, the score was 2.

As many of the patients had a concomitant aphasia, anomic errors such as semantic and phonological errors had to be separated from apraxic errors, so we incorporated Wambaugh and Dabul’s criteria in order to make correct decisions about speech apraxic errors.^14^^,^^15^ Therefore, while scoring, we made differential diagnosis of paraphasias and verbal apraxia errors. The procedure of scoring for this task was time consuming and careful and videos were seen several times.

Total score of verbal apraxia task was 50 representing the maximum error and the highest amount of apraxia (cut-off: 12). Lawsche’s content validity ratio of this task was above 50% for all items according to 25 experts’ opinions, and inter-rater reliability coefficient was 83% (P < 0.001). Cut-off score was determined according to the fifth percentile of 102 healthy adults’ performance (age range of 20–80 years). Cut-off score also was validated by clinical judgment as is described in severity ratings section.

*Severity ratings*

Two certified speech-language pathologists with good experience with adult with apraxia who were blind to lesion information, viewed each videotape and determined the presence or absence of oral and verbal apraxia as well as a severity rating for each.

The five-point equal-appearing-interval scales used to evaluate the severity of oral and verbal apraxia was as follows: 0 = no impairment; 1 = mild; 2 = mild-to-moderate; 3 = moderate-to-severe; and 4 = severe.

The same scores or 1 score difference were considered as total agreement. If there were 2 or more score difference, a third judge was recruited who no longer scored but just accepted one of the two scores. These ratings were used for severity analysis of apraxias, also for validation of cut-off points.

It should be noted that except in one case (whose score in the oral apraxia task was 4 but was estimated without oral apraxia by judges), all cut-off points, which were based on fifth percentile of healthy adults, fully matched to clinical judgments.

*Brain areas*

Three brain areas were selected to study: Broca’s area, left insula, and left MFG. Two former areas being considered because of their history of contributions in speech articulation process and the latter was considered as a control area.

A neuroradiologist who was blind to clinical and demographic profile of patients, rated a yes–no scale for each specified region for each patient according to their CT or MRI scans using Thalairach Atlas of the Brain.

## Results

***Subjects***

Table 1 summarizes clinical and demographic characteristics of subjects. Etiology was not controlled, but as is evident from table 1 was dominated by ischemic stroke.

***Brain areas status in patients with or without oral or verbal apraxia***

Table 2 represents the number of impaired targeted brain areas for each group of patients with or without oral or verbal apraxia.

It is evident from table 2 that the frequency of impairment of Broca’s area and insula for oral apraxics is high and close to each other (insula > Broca’s area) while MFG is lower than both areas. *χ*^2^ revealed that the difference between impaired and spared areas for any three areas is significant (P < 0.05). In patients without oral apraxia the difference of impaired–spared areas are not significant (Broca’s area and insula > MFG).

In patients with verbal apraxia, *χ*^2^ showed a significant difference of impaired–spared areas for Broca’s area and insula (P < 0.05) but not MFG. The frequency of impairments is as follows: insula > Broca’s area > MFG. In patients without verbal apraxia, the frequency of Broca’s area and insula impairment are equal and much higher than MFG.

**Table 1:** Clinical and demographic data

**Variable**	**Age (year)**	**Education (year)**	**Post onset time (month)**	**Etiology**					
	**Mean ± SD**	**Mean ± SD**	**Mean ± SD**	**Ischemic **	**Hemorrhagic **	**Stroke **	**Operated **	**Operated **	**Trauma**
				**stroke**	**stroke**	**(unspecified)**	**tumor**	**AVM**	
Gender									
Female (n = 17)	59.47 ± 17.76	10.82 ± 5.19	33.11 ± 29.48	10	0	4	1	1	1
Male (n = 29)	50.8 ± 13.33	12.58 ± 4.10	20.75 ± 23.53	21	3	1	0	0	4
Total (n = 46)				31	3	5	1	1	5

SD: Standard deviation

**Table 2:** Brain regions involved in groups of with and without apraxia

**Group**	**Number**								
	**Impaired** **Broca’s** **area**	**Spared** **Broca’s** **area**	***χ*** ^2^	**Impaired** **MFG**	**Spared** **MFG**	***χ*** ^2^	**Impaired** **insula**	**Spared** **insula**	***χ*** ^2^
With oral apraxia	32	7	16.02*	26	13	4.33*	35	4	24.64*
Without oral apraxia	5	2	1.28	1	6	3.57	4	3	0.14
With verbal apraxia	30	5	17.85*	25	10	6.42	32	3	24.02*
Without verbal apraxia	7	4	0.81	2	9	4.45*	7	4	0.81

* P < 0.05. MFG: Middle frontal gyrus

**Table 3:** Impaired brain regions in different severities of oral apraxia

**Group**	**Brain region**								
	**Broca’s area** **impairment**		***χ*** ^2^	**MFG impairment**		***χ*** ^2^	**Insula imparment**		***χ*** ^2^
	**Yes**	**No**		**Yes**	**No**		**Yes**	**No**	
Without oral apraxia	5	2	1.28	1	6	3.57	4	3	0.14
Mild	4	4	0.00	5	3	0.50	5	3	0.50
Mild-to-moderate	12	2	7.14*	7	7	0.00	13	1	10.28*
Moderate-to-severe	9	0	5.44*	7	2	2.77	9	0	5.44*
Severe	7	1	4.50*	7	1	4.50*	8	0	4.50*

* P < 0.05. MFG: Middle frontal gyrus

**Table 4:** Impaired brain regions in different severities of verbal apraxia

**Group**	**Brain region**								
	**Broca’s area ** **impairment**		***χ*** ^2^	**MFG impairment**		***χ*** ^2^	**Insula imparment**		***χ*** ^2^
	**Yes**	**No**		**Yes**	**No**		**Yes**	**No**	
Without verbal apraxia	7	4	0.81	2	9	4.45*	7	4	0.81
Mild	4	2	0.66	3	3	0.00	4	2	0.66
Mild-to-moderate	5	2	1.28	3	4	0.14	7	0	5.44*
Moderate-to-severe	15	1	12.25*	14	2	9.00*	15	1	12.25*
Severe	6	0	4.50*	5	1	2.66	6	0	4.50*

* P < 0.05. MFG: Middle frontal gyrus

***Brain areas status in different severities of apraxia***

Tables 3 and 4 compare impaired–spared brain areas in different severities of oral and verbal apraxia respectively.

As is evident from table 3, in mild-to-moderate oral apraxia both Broca’s area and insula are significantly impaired (P < 0.05) while MFG shows indifference. This is also through for moderate-to-severe cases, but here MFG is more impaired than spared. In severe cases three areas are much impaired (insula > Braca’s area and MFG).

It can be seen from table 4 that in mild-to-moderate cases of verbal apraxia, insula is significantly impaired (all seven patients had impaired insula), but Broca’s area and MFG are not significantly impaired (Broca’s area > MFG). In moderate-to-severe and severe cases all three areas are much impaired.

***Comparison of brain regions impairment in different groups***

In order to consider the co-occurrence of two apraxias, we further grouped them into six categories: (1) patients who did not have any form of apraxia (without any apraxia), (2) patients who just had oral apraxia without verbal apraxia (oral apraxia only), (3) patients who just had verbal apraxia without oral apraxia (verbal apraxia only), (4) patients who had both forms of apraxia with identical severity (co-occurred apraxia), (5) patients who had both forms of apraxia with different severities so that oral apraxia was with two or more severity score higher than verbal apraxia (oral apraxia prominent), and (6) patients who had both forms of apraxia with different severities so that verbal apraxia was with two or more severity score higher than oral apraxia (verbal apraxia prominent). Table 5 provides comparison of impaired–spared areas in these subgroups.

**Table 5:** Impaired brain regions in different co-occurred groups

**Group**	**Brain region**								
	**Broca’s area ** **impairment**		***χ*** ^2^	**MFG impairment**		***χ*** ^2^	**Insula ** **impairment**		***χ*** ^2^
	**Yes**	**No**		**Yes**	**No**		**Yes**	**No**	
Without any apraxia	4	2	0.66	1	5	2.66	3	3	0.00
Oral apraxia only	3	2	0.20	1	4	1.80	4	1	1.80
Verbal apraxia only	1	0	-	0	1	-	1	0	-
Co-occurred apraxia	27	3	19.20*	23	7	8.53*	28	2	22.53*
Oral apraxia prominent	1	1	0.00	1	1	0.00	2	0	1.00
Verbal apraxia prominent	1	1	0.00	1	1	0.00	1	1	0.00

* P < 0.05. MFG: Middle frontal gyrus

As is shown in table 5, in oral apraxia only cases, Broca’s area shows a relative homology, while insula

is highly impaired and MFG is highly spared. In the single case of verbal apraxia only, Broca’s area and insula are impaired and MFG is spared. In co-occurred apraxia all three cases are significantly impaired (P < 0.05) (insula > Broca’s area > MFG). In two cases with oral apraxia prominent pattern, insula is impaired, while the other areas show half and half impairment. In two cases with verbal apraxia prominent pattern, three areas show fifty-fifty impairment.

It should be mentioned here that in calculating* χ*^2^, we encountered with the problem of low expected values in some cells, which could not be solved by combining values because of the nature of variables. For interpretation of the results on those situations, we were more relying on the total number of individuals in each subgroup.

## Discussion

This research exploited lesion study to search for possible commonalities between brain areas impaired in patients with oral and verbal apraxia. Regarding our first question on the subject of oral apraxia, the number of impaired–spared brain areas for all three regions in oral apraxic patients was significantly different. But what should be noticed is that the frequencies of involvement of these regions were dissimilar. Broca’s area and Insula were both considerably more impaired in comparison to MFG.

The second question targeted verbal apraxia, and the results showed that the impaired Broca’s area and insula in patients with verbal apraxia were significantly higher than spared areas, but it was not true for MFG. Hence, it is apparent from the results that both Broca’s area and Insula were impaired significantly for both kinds of apraxia.

Concerning the third question which was focused on severity of apraxias, the noticeable finding was seen in mild-to-moderate oral apraxia subgroup, where both Broca’s area and insula were significantly impaired while MFG showed indifference. Furthermore, this was true for moderate-to severe cases, but here impaired–spared areas for MFG were somewhat closer to the other areas. This pattern was changed for verbal apraxia (Table 4), in which Insula was prominently impaired in moderate-to-severe patients but for the other more severe cases, the status of the three areas were somewhat closer to each other.

The relationship of severity of verbal apraxia to extent of brain damage and lesion site has been investigated by Ogar et al.^16^ They found out that mild cases had lesions restricted to the insula and immediately surrounding areas. But more severe cases had lesions encompassing the insula and MFG, with most lesions also involving Broca’s area, the basal ganglia, external capsule, and internal capsule. They proposed that we should regard verbal apraxia as a collection of symptoms related to different brain areas.

We may reason here that instead of considering the most severe forms of apraxia, which raise the possibility of co-occurrence and overlapping, it is better to stick to more moderate forms to better segregate areas responsible for it.

Regarding our forth question in relation to co-occurrence of apraxias, we had the opportunity of looking at more pure forms in patients. In the oral apraxia only group, Insula was significantly impaired while MFG was significantly spared and Broca’s area although was more impaired than spared, the difference was not significant. In the only case with pattern of verbal apraxia only, both Broca’s area and Insula were impaired, while MFG was spared. Also in oral apraxia prominent subgroup, Insula was prominently impaired. Overall as we can infer from table 5, Insula (not Broca’s area) was more evidently impaired in oral apraxia subgroups, and both areas were impaired in verbal apraxia subgroups. The latter result may challenge the results of Hillis et al.^8^ that proved Broca’s area, but not Insula to be associated strongly with verbal apraxia. Likewise, our results regarding verbal apraxia is neither consistent with Richardson et al.^17^ conclusion that damage to the posterior portion of Broca’s area is a better predictor of AOS than insula involvement nor with Ogar et al.^16^ and Dronkers^7^ who found that insula damage is a more reliable predictor of motor speech impairment compared to Broca’s area involvement. It can be seen from our results that both Broca’s area and Insula are involved in verbal apraxia.

On the other hand, when we consider our results for both forms of apraxia, Insula was conspicuously impaired in oral and verbal apraxia and different severities and prominent forms of both apraxias. This finding is in line with literature that view Insula as a main cite of damage in some case reports of verbal apraxia,^5^^,^^7^ and as one of the impaired areas mentioned for oral apraxia.^18^

The last question of the present research regards the commonalities of neuropathology of oral and verbal apraxia. Ackermann and Riecker^5^ concluded that no strict co-occurrence of verbal apraxia and oral apraxia in subjects within trasylvian pathology seems to occur.^5^ This seems to be the case with our results because despite great correlations, no one-to-one correspondence was seen in clinical or neuroimaging profile of the patients. Yet, integrating all the results may reveal Insula as the common area involved in both apraxias of this research.

It seems that the most important finding of the present research is that examining neural correlates of apraxia is better performed with milder forms. For future investigations, it seems reasonable that we should not seek for absolute frequencies in calculation of brain areas impaired in a neuropsychological disorder, but looking for relative frequencies as has been applied in this study. Another way of answering the questions of this research may be searching for pure types of apraxia in milder forms and comparing their brain areas involved.

Finally, the results of this study should be considered cautiously because of the limitations which we recommend to be controlled in future studies. We did not control the age range of the subjects. Furthermore, the post-onset time was just controlled for the acute phase not for the evolutionary stage of spontaneous recovery.

## Conclusion

Based on the results of this study, we may conclude that Broca’s area and left Insula were impaired above chance in comparison to MFG in both verbal and oral apraxic patients, and Insula was more prominently impaired. Also it is concluded that milder forms of apraxia are better candidates for identification of involved brain areas.
